# Orbital Extension of Sinonasal Mucocele Presenting as Proptosis: A Report of Two Cases

**DOI:** 10.7759/cureus.111489

**Published:** 2026-06-25

**Authors:** Mohamed Boualane, Youssef Chajire, Zakaria Azemour, Nabil Bouslous, Moustaine M Omar

**Affiliations:** 1 Ophthalmology, Centre Hospitalier Universitaire Mohammed VI, Agadir, Agadir, MAR

**Keywords:** computed tomography, diplopia, frontoethmoidal mucocele, magnetic resonance imaging, orbital complications, orbital extension, proptosis, sinonasal mucocele

## Abstract

Sinonasal mucoceles are benign cystic lesions caused by obstruction of sinus drainage; however, they may progressively expand and erode surrounding bone, leading to orbital complications.

We report two cases of sinonasal mucoceles with orbital extension presenting as proptosis. The first case involved a 34-year-old man presenting with acute proptosis, diplopia, and decreased visual acuity, with imaging revealing a frontoethmoidal lesion extending into the orbit. The second case involved a 43-year-old woman with progressive proptosis without visual impairment, also related to a frontoethmoidal mucocele. Management differed between the two cases, with spontaneous regression in the first and surgical treatment in the second, both resulting in favorable outcomes.

These cases illustrate the variable clinical presentation of sinonasal mucoceles and highlight the importance of imaging in diagnosis and management. Early recognition and appropriate treatment are essential to prevent visual complications.

## Introduction

Sinonasal mucoceles are benign, expansile cystic lesions resulting from obstruction of the sinus ostia and retention of mucus within the paranasal sinuses [1,2]. Progressive accumulation of mucus may cause expansion of the affected sinus with bone remodeling or erosion and extension into adjacent structures, particularly the orbit and, less commonly, the intracranial cavity [2,3]. Orbital involvement may result in proptosis, diplopia, restricted ocular motility, and visual impairment [1,4].

The frontal and ethmoidal sinuses are the most commonly affected sites. Frontal mucoceles account for approximately 60-65% of cases, followed by ethmoidal mucoceles (20-30%), whereas maxillary and sphenoidal localizations are less frequent [3,5]. Clinical presentation depends on the location and extent of the lesion. Imaging plays a central role in diagnosis and treatment planning. Computed tomography is useful for evaluating bony erosion, and coronal reconstructions are particularly valuable for assessing orbital roof and medial wall involvement and for surgical planning. Magnetic resonance imaging provides better assessment of soft tissue, orbital, and intracranial extension [1,3].

Early recognition and appropriate management are essential to prevent irreversible visual complications. This report highlights the diagnostic challenges and clinical variability of frontoethmoidal mucoceles with orbital extension and describes an unusual case of spontaneous regression associated with complete visual recovery, a rarely reported outcome in the literature.

## Case presentation

This retrospective case series was conducted at the Department of Ophthalmology, Mohammed VI University Hospital, Agadir, Morocco. It includes two patients with frontoethmoidal sinonasal mucoceles presenting with orbital extension and proptosis, identified between January 2022 and December 2023. Clinical, ophthalmologic, radiologic, therapeutic, and follow-up data were retrospectively reviewed. The cases were not selected consecutively but were included because they illustrate two distinct clinical presentations and outcomes of the same condition.

Case 1

**Figure 1:**
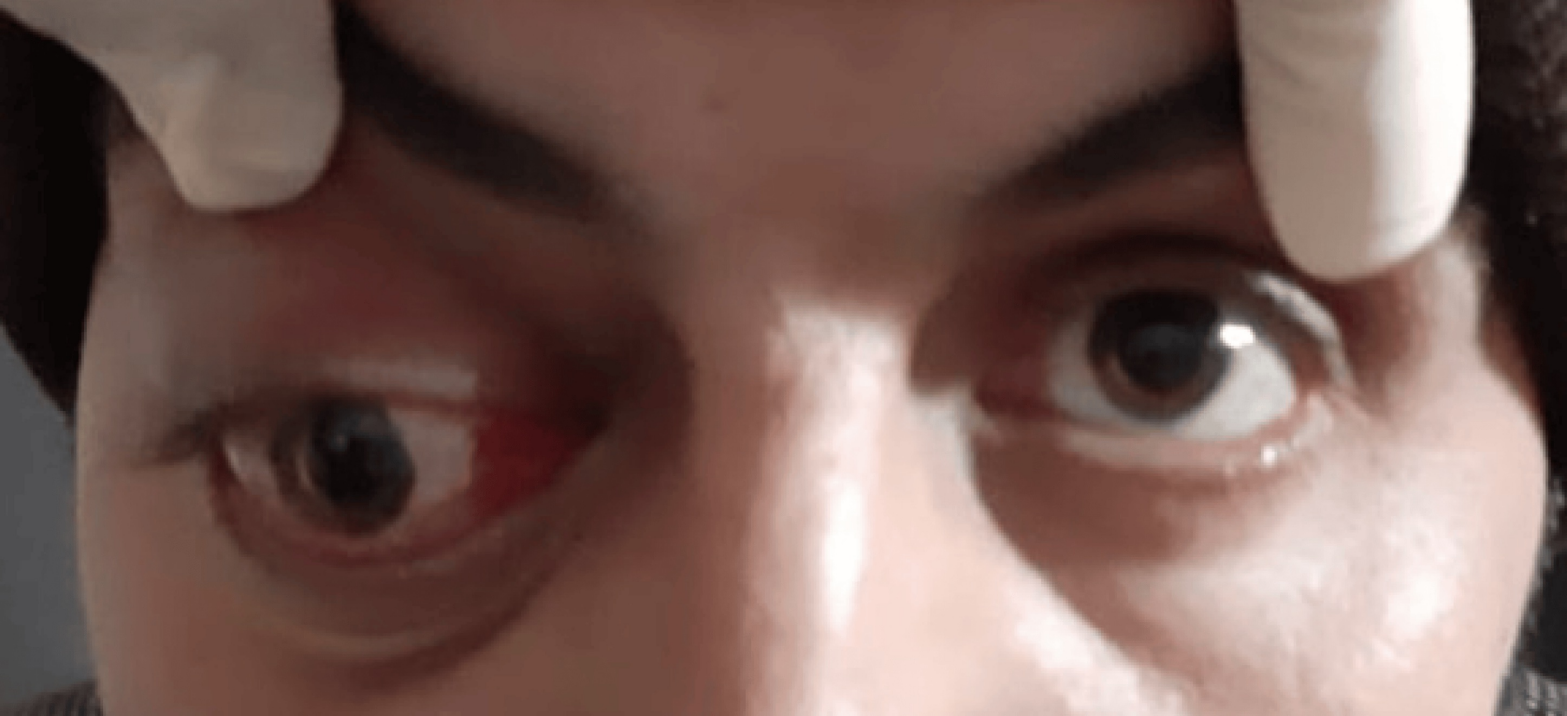
Eyelid edema associated with conjunctival chemosis and non-axial proptosis of the right eye.

A 34-year-old man with a history of allergic rhinitis and no history of facial trauma, sinonasal surgery, diabetes, or immunosuppression presented with a one-week history of acute right-sided proptosis associated with binocular diplopia and decreased visual acuity in the right eye. He reported progressive swelling of the right eyelid and ocular discomfort but no fever, purulent nasal discharge, or systemic symptoms. Ophthalmologic examination revealed right non-axial, non-pulsatile proptosis with inferolateral displacement of the globe, associated with right upper eyelid edema and conjunctival chemosis (Figure 1).

**Figure 2:**
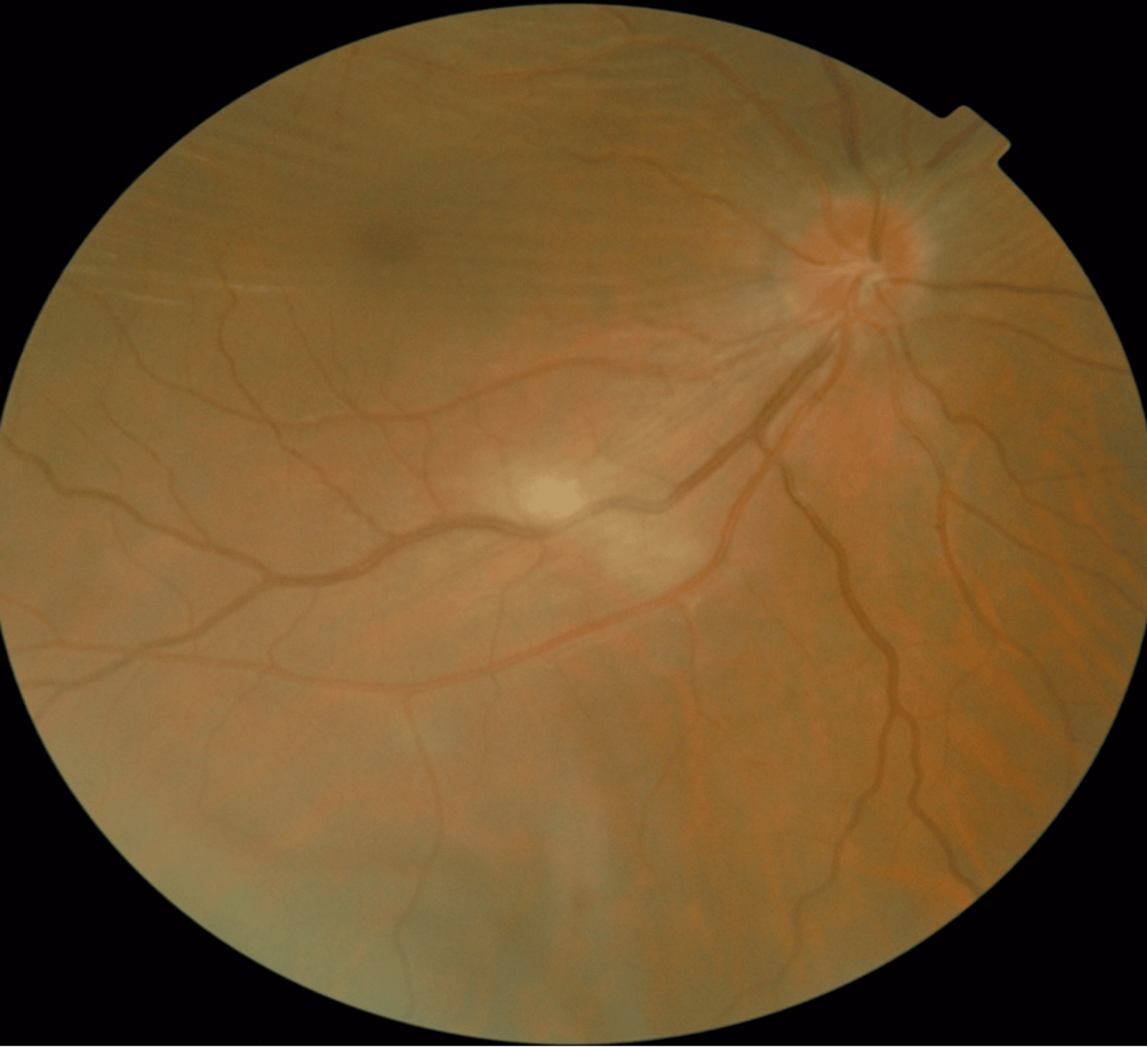
Grade 2 optic disc edema of the right eye with associated papillomacular retinal folds.

Best-corrected visual acuity was 3/10 in the right eye and 10/10 in the left eye. Ocular motility of the right eye was restricted in all directions of gaze, without pain during ocular movements. Pupillary reflexes were normal and symmetrical, with no evidence of a relative afferent pupillary defect. Intraocular pressure measured 23 mmHg in the right eye and 19 mmHg in the left eye. Fundus examination of the right eye showed marked optic disc edema associated with retinal folds in the papillomacular region (Figure 2), while the left eye was unremarkable.

Given the acute inflammatory presentation with eyelid edema and chemosis, orbital cellulitis was initially considered in the differential diagnosis. Other differential diagnoses included a subperiosteal abscess and an orbital mass lesion. Empirical intravenous ceftriaxone (2 g/day) and metronidazole (500 mg every eight hours) were administered for 48 hours pending radiologic evaluation.

**Figure 3:**
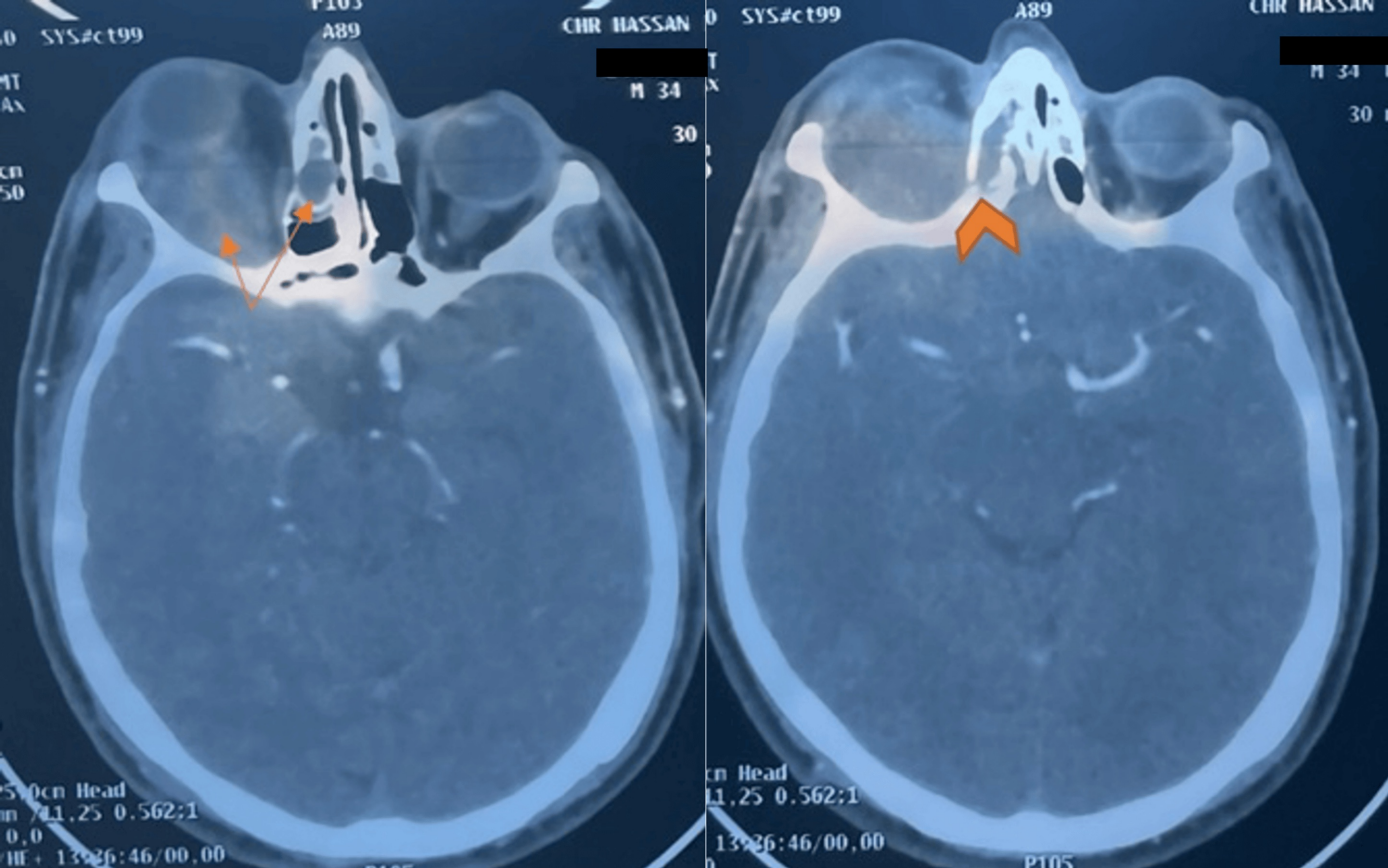
Facial CT scan (axial view) showing an ethmoido-orbital fluid-filled lesion (arrow) with erosion of the medial orbital wall (arrowhead). White arrow indicates the ethmoido-orbital fluid-filled lesion (mucocele). White arrowhead indicates erosion of the medial orbital wall.

Non-contrast computed tomography of the paranasal sinuses subsequently demonstrated a right frontoethmoidal expansile cystic lesion extending into the orbit with inferolateral displacement of the globe and erosion of the orbital roof and medial wall, without evidence of intracranial extension (Figure 3).

**Figure 4:**
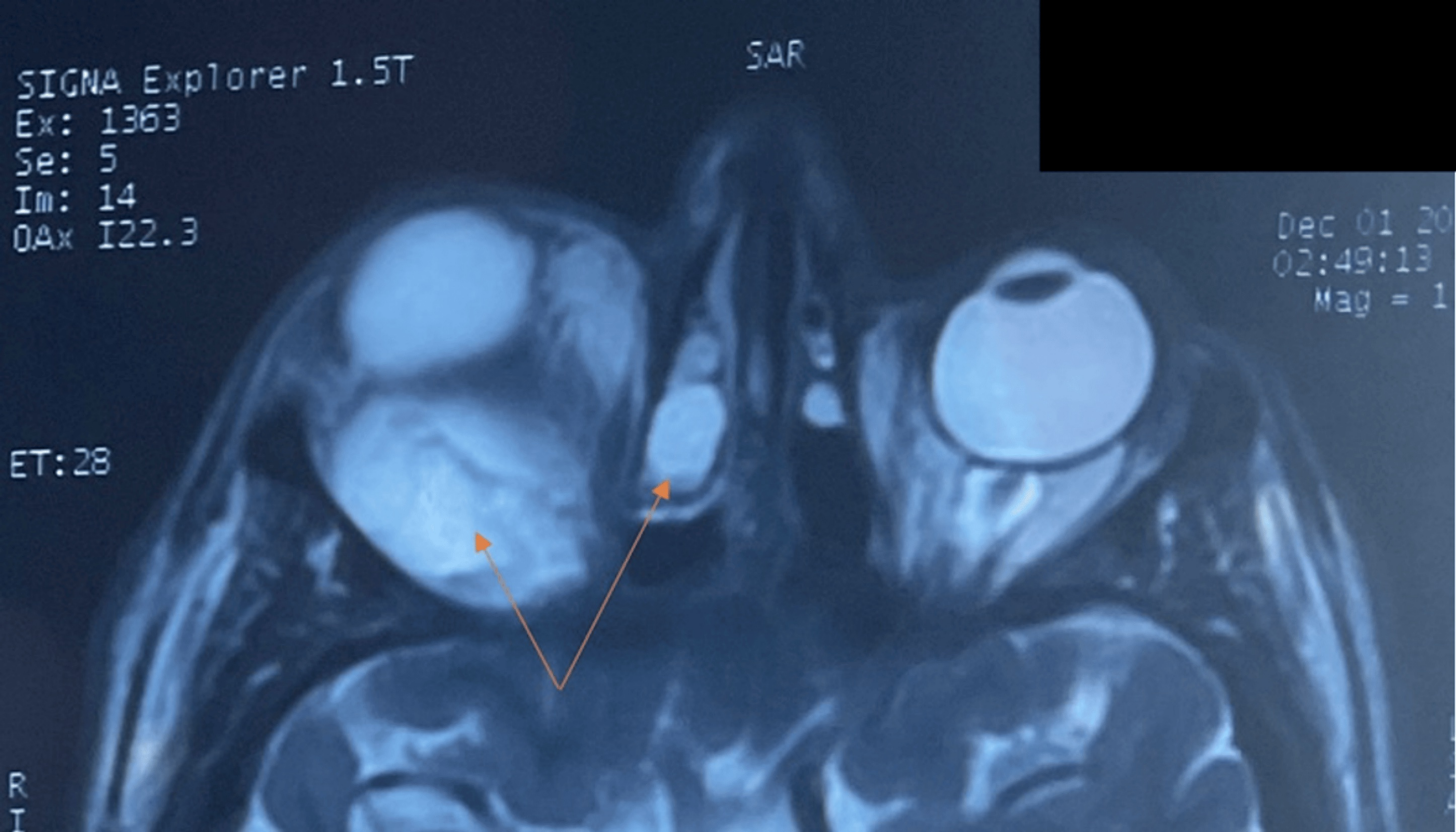
Axial T2-weighted facial MRI showing a well-circumscribed expansile ethmoido-orbital lesion (arrow) displacing the globe laterally.

Magnetic resonance imaging confirmed the diagnosis of sinonasal mucocele with orbital extension and showed no intracranial extension or dural involvement (Figure 4). 

**Figure 5:**
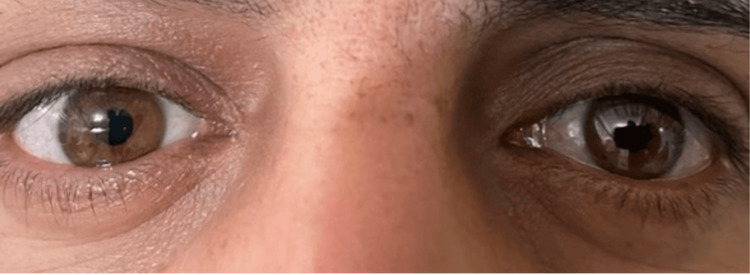
Normal appearance of the right globe and eyelid one month later.

Based on the imaging findings and the absence of clinical or radiological evidence of orbital cellulitis, antibiotic therapy was discontinued. The patient was scheduled for endoscopic endonasal marsupialization and drainage of the frontoethmoidal mucocele in order to decompress the orbit and restore normal sinus drainage. However, no surgical intervention was ultimately performed because the patient was lost to follow-up before the planned procedure. He returned one month later with spontaneous progressive regression of proptosis and complete recovery of visual acuity to 10/10 (Figure 5).

Ophthalmologic examination at presentation demonstrated normal ocular motility, resolution of eyelid edema and chemosis, and a normal fundus examination with complete resolution of optic disc edema and papillomacular retinal folds. No recurrence has been observed during subsequent follow-up.

Case 2

**Figure 6:**
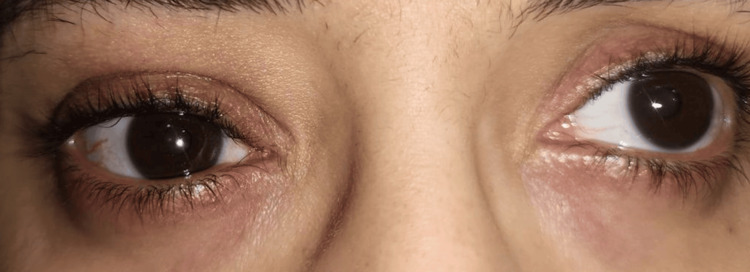
Unilateral axial proptosis of the right eye.

A 43-year-old woman with no significant medical history presented with a three-month history of progressive right-sided proptosis. She denied diplopia, ocular pain, decreased vision, fever, or other systemic symptoms. Visual acuity was preserved at 10/10 in both eyes. Examination revealed axial, non-pulsatile, non-reducible proptosis of the right eye without associated inflammatory signs. Ocular motility was full in all directions of gaze, intraocular pressure was within normal limits, and fundus examination was unremarkable (Figure 6). The left eye was normal.

**Figure 7:**
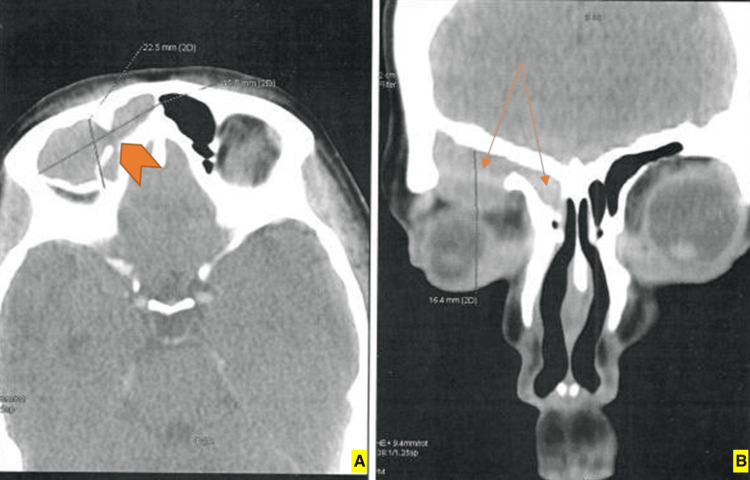
Computed tomography findings. (A) Coronal CT image demonstrating erosion of the anterior portion of the orbital roof (arrowhead). (B) Axial CT image showing an ethmoido-orbital fluid-filled lesion (arrow) with orbital extension. White arrow indicates the ethmoido-orbital fluid-filled lesion (mucocele). White arrowhead indicates erosion of the anterior orbital roof.

The differential diagnosis included orbital tumors, thyroid-associated orbitopathy, and sinonasal lesions with orbital extension. Computed tomography demonstrated a right frontoethmoidal mucocele with intraorbital extension through a bony defect of the anterior orbital roof consistent with pressure-related bone erosion secondary to chronic expansion of the mucocele (Figure 7).

Given the frontal location and extent of the lesion, the patient underwent surgical drainage and wide marsupialization through an external frontal approach under general anesthesia. The mucocele contents were evacuated and adequate drainage was achieved. No intraoperative or postoperative complications occurred.

Postoperative recovery was uneventful, with complete regression of proptosis and preservation of visual acuity. Follow-up examinations remained normal, with no evidence of recurrence after more than one year of follow-up.

## Discussion

Sinonasal mucoceles are benign, slowly expanding lesions caused by obstruction of sinus drainage, leading to progressive mucus retention and enlargement of the affected sinus cavity [1-3]. Although histologically benign, they may cause bone remodeling and erosion, allowing extension into adjacent structures, particularly the orbit. This explains why some patients initially present to ophthalmology rather than otolaryngology services.

The frontal sinus is the most frequently involved site, followed by the ethmoidal sinus. Fronto-ethmoidal lesions are more likely to produce orbital manifestations because of their close anatomical relationship with the orbital roof and medial wall [2,5]. Orbital involvement may result in proptosis, diplopia, restricted ocular motility, eyelid edema, chemosis, increased intraocular pressure, and visual impairment secondary to optic nerve compression [6]. This was illustrated in Case 1, where optic disc edema and decreased visual acuity were present at presentation.

Clinical findings suggestive of imminent visual compromise include decreased visual acuity, optic disc edema, relative afferent pupillary defect, and radiologic evidence of optic nerve compression. Early recognition of these features is important because delayed treatment may result in irreversible visual loss [6,7].

Our two patients illustrate the variable clinical spectrum of this condition. The first case presented acutely with unilateral proptosis, diplopia, optic disc edema, and decreased visual acuity, whereas the second case had slowly progressive proptosis with preserved vision. Acute inflammatory presentations may mimic orbital cellulitis and delay diagnosis if sinonasal pathology is not considered, as illustrated in our first case [4,8].

The differential diagnosis includes orbital cellulitis, subperiosteal abscess, and orbital tumors. Orbital cellulitis and abscesses are typically associated with fever, inflammatory signs, and laboratory abnormalities, whereas orbital tumors generally present as solid masses on imaging. In contrast, mucoceles are characterized by expansile cystic lesions associated with sinus opacification and bony remodeling [3,8,9]. When unilateral proptosis is associated with sinonasal opacification on imaging, early otolaryngology referral should be considered to facilitate prompt diagnosis and appropriate management.

Spontaneous regression of a frontoethmoidal mucocele with orbital extension is uncommon. A possible explanation in our patient is spontaneous drainage of the mucocele into the nasal cavity through partial rupture of its wall or decompression through areas of bone erosion, resulting in a reduction of intralesional pressure and orbital mass effect. A similar mechanism has been described in spontaneous drainage of an ethmoidal mucocele [10].

Imaging is essential for diagnosis and treatment planning. Computed tomography is particularly useful for identifying sinus opacification, bony erosion, and orbital extension, while magnetic resonance imaging better characterizes the cystic nature of the lesion, evaluates locoregional extension, and helps differentiate mucoceles from associated expansile soft tissue processes [1,3,8]. In both cases, CT imaging identified bony erosion and orbital extension, while MRI provided additional information regarding adjacent orbital soft tissue involvement. These findings were important for surgical planning and assessment of the relationship between the lesion and adjacent orbital structures.

Management is primarily surgical and aims to restore sinus drainage, decompress adjacent structures, and prevent recurrence. Endoscopic marsupialization is currently preferred in many cases because it is minimally invasive and associated with favorable outcomes [1,4]. External or combined approaches may still be required for selected frontal lesions. In our second case, the location and extent of the frontal component prompted the use of an external approach, which allowed adequate exposure and drainage, resulting in complete regression of proptosis. The spontaneous improvement observed in the first patient was unusual and should not replace standard management when visual function is threatened.

Recurrence rates after surgery are generally low, with most published series reporting rates below 10%. Recurrence may vary according to lesion location and the surgical approach used (endoscopic, external, or combined). However, delayed recurrence several years after treatment has been described, supporting the need for long-term follow-up. Orbital extension may also be associated with a higher risk of recurrence, possibly because extensive lesions may be more difficult to completely drain and may involve more complex anatomical regions [5].

Prognosis may vary according to lesion location. Anterior mucoceles involving the frontal or maxillary sinuses usually have a more favorable outcome, whereas posterior lesions, particularly sphenoidal and posterior ethmoidal mucoceles, carry a greater risk of visual and neurological complications because of their proximity to the optic nerve, orbital apex, and adjacent cranial neurovascular structures [5,6]. Both cases reported in the present study correspond to anterior frontoethmoidal lesions and were associated with favorable outcomes.

Visual prognosis is generally good when diagnosis and treatment are prompt. However, urgent decompression may be required in the presence of rapidly progressive visual loss, optic disc edema, relative afferent pupillary defect, or imaging evidence of optic nerve compression. Delayed management may result in permanent optic neuropathy or persistent ocular motility disorders [6,7]. Therefore, sinonasal mucoceles should be included in the differential diagnosis of unilateral proptosis, and multidisciplinary collaboration between ophthalmologists, otolaryngologists, radiologists, and neurosurgeons when skull base or intracranial extension is suspected is essential for optimal management.

## Conclusions

Sinonasal mucocele is an uncommon benign cystic lesion that may become clinically significant when orbital extension occurs. Our two cases illustrate the variable clinical presentation of this condition, ranging from acute inflammatory proptosis with visual impairment to slowly progressive proptosis with preserved visual function. The unusual spontaneous regression observed in one patient highlights the variability of clinical evolution but should not replace standard surgical evaluation when visual function is threatened. Early diagnosis, appropriate imaging assessment, and multidisciplinary management remain essential to prevent visual complications and optimize outcomes.
